# Effects of ploidy level and haplotype on variation of photosynthetic traits: Novel evidence from two *Fragaria* species

**DOI:** 10.1371/journal.pone.0179899

**Published:** 2017-06-23

**Authors:** Song Gao, Qiaodi Yan, Luxi Chen, Yaobin Song, Junmin Li, Chengxin Fu, Ming Dong

**Affiliations:** 1 Key Laboratory of Conservation Biology for Endangered Wildlife of the Ministry of Education, and College of Life Sciences, Zhejiang University, Hangzhou, China; 2 Research Institute of Zhejiang University-Taizhou, Taizhou, China; 3 Zhejiang Provincial Key Laboratory of Plant Evolutionary Ecology and Conservation, Taizhou University, Taizhou, China; 4 Key Laboratory of Hangzhou City for Ecosystem Protection and Restoration, and College of Life and Environmental Sciences, Hangzhou Normal University, Hangzhou, China; University of the Chinese Academy of Sciences, CHINA

## Abstract

To reveal the effects of ploidy level and haplotype on photosynthetic traits, we chose 175 genotypes of wild strawberries belonging to two haplotypes at two types of ploidy levels (diploidy and tetraploidy) and measured photosynthetic traits. Our results revealed that ploidy significantly affected the characteristics of light-response curves, CO_2_-response curves, and leaf gas exchange parameters, except intercellular CO_2_ concentration (*C*_i_). Tetraploid species had a lower light saturation point (*LSP*) and CO_2_ saturation point (*CSP*), higher light compensation point (*LCP*), dark respiration (*R*d), and CO_2_ compensation point (*CCP*) than diploid species. Furthermore, tetraploid species have lower photosynthetic capacity than diploid species, including net photosynthetic rate (*P*_n_), stomatal conductivity (*G*_s_), and transpiration rate (*T*_r_). In addition, haplotype had a significant effect on *LSP*, *CSP*, *T*_r_, and *C*_i_ as well as a significant interactive effect between ploidy and haplotype on the maximal photosynethic rate of the light-response curve and *R*_d_. Most of the variance existed within haplotypes among individuals. These results suggest that polyploidization was the main driver for the evolution of photosynthesis with increasing ploidy level (i.e. from diploidy to tetraploidy in *Fragaria* species), while the origin of a chromosome could also affect the photosynthetic traits and the polyploidization effect on photosynthetic traits.

## Introduction

Polyploidy is a prevalent biological phenomenon of the chromosomal evolution of extant species and genera, including major crop plants such as rice, maize, wheat, soybean, and cotton [1, 2]. Most, if not all plant species have a polyploidy ancestry [3], and polyploidy may have been critically important for flowering plant diversification and the development of cytological, morphological, chemical, physiological, and molecular characteristics [4–7], and subsequently the interactions with other members of the biotic community [5, 8]. Exploring the ecological effects of polyploidy for key traits of plant species will help to elucidate the evolutionary significance of polyploidization and potential for its occurrence [5].

Most of the previously published ecological studies focused on the effects of different cytotypes on plants traits, i.e. the number of chromosomes [9, 10]. Among these studies, advantages and disadvantages of allopolyploids as well as autoployploids have often been compared [9–11]. Photosynthesis is a central metabolic process in plants [12, 13] and photosynthetic traits can be used as indicators to judge the adaptability and resistance of plants [14]. The photosynthetic rate is sensitive to the ploidy level, which correlates with the amount of DNA per cell [15, 16] and has been well documented in numerous species [17, 18]. Both positive and negative correlations with ploidy level have been reported [16, 19, 20], and the anatomy structure as well as biochemical pathways have been well studied [15]. Recently, following the realization that genotypes may affect the resulting gene expression after polyploidization, Oates et al. reported that the effect of induced polyploidy on fertility and morphology of allotetraploids and autotetraploids of *Rudbeckia spp*. were variable both among and within genotypes [21]. The genotype is part (DNA sequence) of the genetic makeup of a cell), and therefore of an organism or individual, thus determining a specific characteristic (phenotype) of that cell/organism/individual. Polyploidization can result in structural changes of the genome, increase the genetic diversity by producing new genotypes through recombination, and enhance the colonization ability in new environments [11]. A haplotype (haploid genotype) is a group of genes in an organism that have been inherited together from a single parent, thus determining the parent-of-origin effect [22]. Furthermore, some haplotypes were verified to be very stable during evolution from diploid to tetraploid and hexaploid, such as in wheat species; consequently, polyploidization had no detectable consequences on the structure and evolution of both haplotypes [23]. We hypothesized that if the haplotype of a plant with different ploidy level were identical (i.e. sharing the same ancestry and no genome exchanges after polyploidization), then the effect of polyploidization should be the main driver of specialization of plants. However, to our knowledge, no other study focused on how haplotypes affect the effect of polyploidization on phenotypic traits and their interactive effect.

*Fragaria* species are stoloniferous perennial herbs that share the same basic architecture and life history with ploidy levels varying from diploidy to decaploidy [24]. As two representative species of strawberry plants, the diploid *Fragaria pentaphylla* and the tetraploid *Fragaria moupinensis* sharing similar haplotypes have a wide and overlapping area of distribution and similar habitats [25]. They provide a good model to test the effect of ploidy and haplotype on the variation of phenotypic traits. For this study, we chose 175 genotypes of wild strawberries that belong to two haplotypes at two levels of ploidy (diploidy and tetraploidy) and we obtained photosynthetic parameters including light-response and CO_2_-response curves as well as gas exchange parameters. We thus revealed the effects of the ploidy and haplotype on the photosynthetic traits and also their interactive effects.

## Materials and methods

### Plant species

*Fragaria pentaphylla* is a wild and diploid (2n = 14) species from the *Fragaria* genus, belonging to the Rosaceae family [26]. It is a perennial herb with a monopodial form of growth. Two small accessory leaflets are often present and plants feature a total of five leaflets. The leaflets are typically not overlapping when pressed and the sepals are spreading. The plant is a hermaphrodite and the fruits can be either red or white. *F*. *pentaphylla* is native to the Chinese provinces of Sichuan Qinghai, Gansu Shanxi, and Henan. This species is most often found in forests, forest clearings, scrub, mountain meadows, and open gravel at elevations between 1000 and 2700 m [26].

*Fragaria moupinensis* is a wild and tetraploid (2n = 28) species from the *Fragaria* genus, belonging to the Rosaceae family [26]. It is also a perennial herb with a monopodial form of growth. Two small accessory leaflets are either present or absent and the Leaflets are typically not overlapping when pressed and sepals always clasp the mature fruit. It is a dioecious plant with red fruits. *F*. *moupinensis* is native to the Chinese provinces of Sichuan, Gansu, Shanxi, Yunnan, and Xizang and is most often found in forest undergrowth, mountain meadows, and grasslands at elevations between 1400 and 4000 m [26].

### Sample collection

During July 2013, we collected seeds and leaves of *F*. *pentaphylla* and *F*. *moupinensis* populations in Gansu, Qinhai, Yunnan, Sichuan and Shanxi Province and Xizang (Tibet) Autonomous Region. All the information for the sampling sites was listed on S1 Table. The field sites did not involve any endangered or protected species, and none of the populations were privately owned or under nature protection. No specific permissions were required for these locations. One to eight (with the distance at least 10 m apart) fruiting plants were randomly selected in every population. In totally, 175 plants from 65 populations were collected (S1 Table). Leaves were quickly dried via silica gel before returning them to the laboratory. Each fruit was squeezed on a filter paper to remove the flesh. Seeds were air-dried before returning to the laboratory and subsequently stored at -20°C.

### Identification of haplotype

During November 2013, we extracted total genomic DNA from dried leaves via a plant total genomic DNA extraction kit (Dingguo Inc, Shang, China). Having examined the published *Fragaria* chloroplast genomes, we found that the NADH dehydrogenase subunit A (ndhA) intron was the most variable non-coding plastid sequence, which was verified to effectively discriminate the haplotype of plants [27]. Primers were designed according to published the ndhA intron sequence of *Fragaria* chloroplast genomes and PCR was conducted according to regular protocols. BioSune Biotechnology Co. Ltd., Shanghai, China, conducted PCR product sequencing. The haplotypes of the individuals were determined via alignment of the ndhA sequence by Aaron Liston from the Oregon State University. We selected haplotype A and haplotype B for the relative population number. The ndhA sequence of haplotype A and haplotype B is presented in the supplemental S1 Fig.

### Identification of ploidy level

We utilized dried leaf tissue (approximately 20 mg) to determine the ploidy level via flow cytometry, using a modified method of Suda and Trávníček [28]: We suspended leaf tissue in 1 mL ice-cold extraction buffer and co-chopped the leaf with a plastic petri dish over a chilled brick with a fresh razor blade for 2 min until arriving at a fine slurry. Large leaf debris was removed via a 600-mesh nylon filter (Shanghai Aoran Hardware market, Shanghai, China). We added 1 ml of pre-cooled extraction buffer to the dish to rush the chopped tissue and we repeated the whole procedure with remaining tissue. We gathered both parts of the supernatant and placed them into a 5 ml centrifuge tube, adding RNaseA solution and incubating at 4°C for 10 min. We then added propidium iodide staining solution to the tube and incubated at 4°C for further 30 min in dark. We utilized an Attune flow cytometer (ThermoFisher Scientific Inc. Waltham, MA, USA) to analyze the stained solutions until 20,000 events were captured. We used the cultivated octoploidy strawberry line ‘Hongyan’ (obtained from Shaojiadu Cooperation of Linhai City, Zhejiang Province, China) as an external standard and inferred the ploidy level from the relative PI value compared to the external standard.

### Seeds germination and transplanting

In total, we chose 97 diploidy genotypes (*F*. *penthaphylla*) and 20 tetraploidy genotypes (*F*. *moupinensis*) that belong to haplotype A, 28 diploidy genotypes (*F*. *penthaphylla*) and 30 tetraploidy genotypes (*F*. *moupinensis*) that belong to hayplotype B for this experiment. During November 2014, four seeds from every *Fragaria* individual were germinated in a walk-in growth chamber with conditions of 22°C during 16 hours of daytime and 15°C during 8 hours of nighttime, with a relative humidity of 90%. 30 days after germination, one seedling (genotype) per haplotype was transplanted and moved to a greenhouse with identical conditions. We randomly selected 20% seedlings and individuals with ambiguous ploidy level to double check the ploidy level by measuring the karyotype via the root-tip squashing method, modifying the method by Nathewet et al. [29]. We harvested approximately 2 cm root tips and pretreated these with 2 mM 8-hydroxyquinoline at room temperature (22°C) for 1 h, followed by keeping them at 4°C for more than 15 h, fixing them in Farmer’s solution for 2 h, soaking them in 1N HCl at 22°C for 1 h, macerating them in 1 N HCl at 60°C for 11 min, and staining them with Carbol fuchsin staining solution for 15 min. Then, we detected the number of chromosomes number using a BA310 digital microscope (Motic Instruments Inc., Xiamen, Fujian, China) at 100 × magnification.

### Measurement of photosynthetic traits

During August 2015, we conducted *in situ* photosynthetic trait measurements on a sunny day on mature middle leaflet at the same position, using the Li-6400XT portable photosynthesis system (LI-COR Biosciences Inc., Lincoln, NE, USA). We used a red-blue LED light source attached to the system to produce steady photosynthetic active radiation (PAR).

To construct the light response curves, we obtained all photosynthesis measurements between 09:30 and 11:00 h (Beijing time) on mature middle leaflet from each plant with a leaf temperature of 25°C, a CO_2_ concentration of 400 ppm and a relative humidity of 70%. A photosynthetically active radiation lamp provided the light source. Prior to the measurements, we allowed the mature middle leaflet to acclimate under a PAR of 2,000 μmol m^-2^ s^-1^ for 30 min to avoid photo-inhibition. As soon as the value stabilized, we exposed the leaves to a series of PAR values for 3 min or so in the following order: 2,000, 1,500, 1,200, 1,000, 800, 600, 400, 200, 100, 50, 20, and 0 μmol m^-2^ s^-1^. The temporal interval between each concentration was 3 min. We fitted the entire photosynthetic light response curves in Origin 8.0 as a binary linear equation [30] calculating the maximum of the net photosynthetic rate (*P*_n_) as *P*_max_. We utilized the following definitions: the light intensity that leads to maximum *P*_n_ was defined as the light saturation point (*LSP*), the light intensity that leads to zero *P*_n_ was defined as the light compensation point (*LCP*), and the *P*_n_ that leads to maximum a PAR of zero was defined as the dark respiration point (*R*_d_). We measured three seedlings per haplotype.

To construct the CO_2_ response curves, we obtained all photosynthesis measurements between 09:30 and 11:00 h (Beijing time) on mature middle leaflet from each plant with a leaf temperature of 25°C, a PAR of 1,000 μmol m^-2^ s^-1^, and a relative humidity of 70%. A small portable cylinder that was filled to a specified CO_2_ pressure supplied the CO_2_. Prior to the measurements, we allowed the mature middle leaflet to acclimate under a PAR of 1,500 μmol m^-2^ s^-1^ for 30 min to avoid photo-inhibition. As soon as the value stabilized, we exposed the mature middle leaflet to a series of CO_2_ concentrations for 5 min or so in the following order: 1,500, 1,200, 1,000, 800, 600, 400, 200, 150, 120, 100, 80, and 50 μmol mol^- 1^. The temporal interval between each concentration was 5 min. We fitted the entire photosynthetic CO_2_-response curves in Origin 8.0 as a binary linear equation and calculated the maximum net photosynthetic rate (*P*_n_) as *P*_max_ [30]. The CO_2_ concentration that leads to maximum *P*_n_ was defined as the CO_2_ saturation point (*CSP*). The CO_2_ concentration that leads to zero *P*_n_ was defined as the CO_2_ compensation point (*CCP*). We measured three seedlings per haplotype.

After obtaining *LSP* of the strawberry (1000 μmol photons·m^–2^·s^–1^ or so), we measured the leaf gas exchange parameters of all 175 genotypes between 08:30 am and 11:30 am. We recorded *P*_n_, stomatal conductance (*G*_s_), transpiration rate (*T*_r_), and intercellular CO_2_ concentration (*C*_i_) at two-hour intervals, choosing three matured leaves per plant (genotype), and performing six consecutive measurements [31].

### Statistical analyses

The data are shown as mean ± standard deviation. We utilized two-way ANOVA to test the effect of ploidy and haplotype on the photosynthetic characteristics of plants, using ploidy and haplotype as the fixed factor. Levene's test was used to evaluate the homogeneity of variance. Reciprocal transform of *P*_n_, *G*_s_, and *T*_r_ were used for ANOVAs analysis, for the variance is not homogeneous. Linear contrasts after ANOVAs were used to analyze the significance of the differences of photosynthetic characteristics between diploidy and tetraploidy in the same haplotype. The existence of variance among ploidies and haplotypes were estimated via VARCOMP analysis [32, 33]. We conducted all these analyses via SPSS 19.0 software and used the software Origin 8.0 for mapping.

## Results

### Characteristics of light-response curves

**Fig 1:**
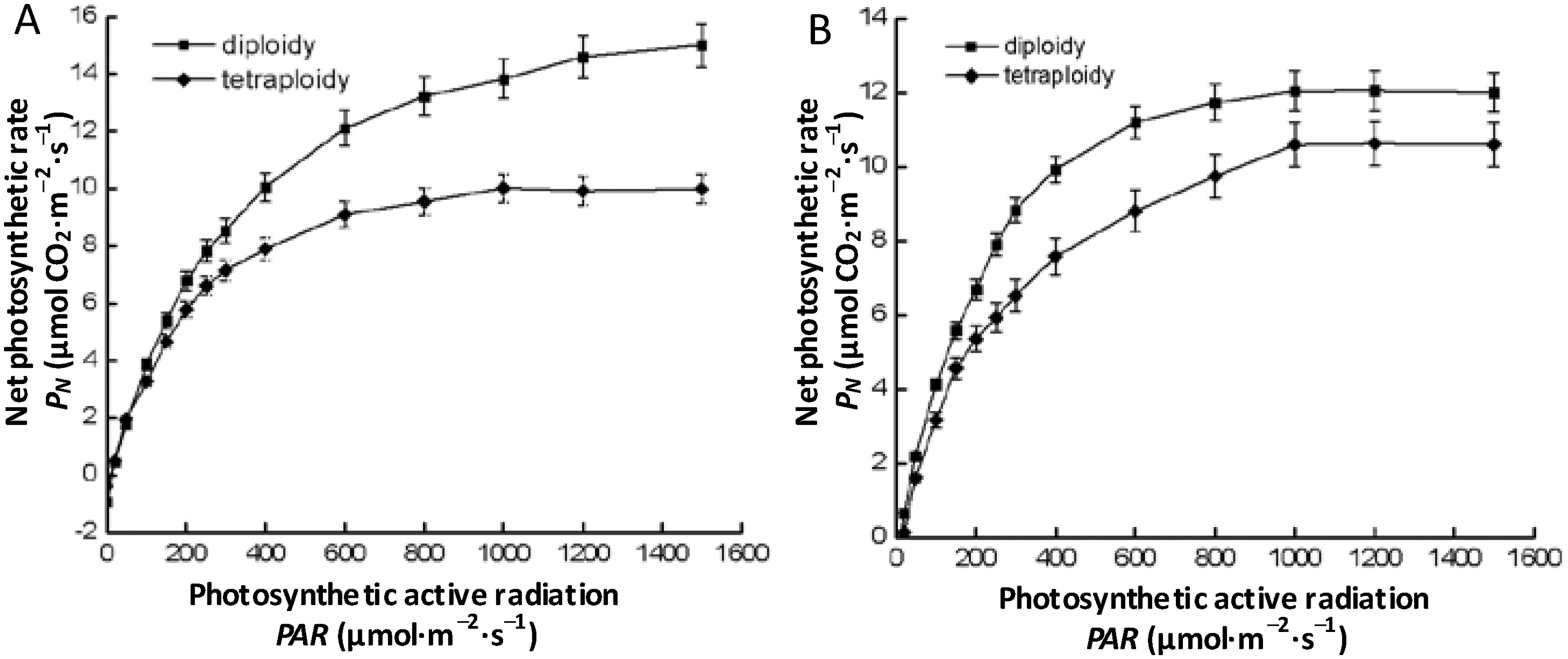
Light response curve of *Fragaria* plants with different ploidy levels of haplotype A (A) and haplotype B (B).

The patterns of light-response curves differed among different ploidy levels as well as among different haplotypes (Fig 1A and 1B, S2 Table). For identical haplotypes, tetraploid species had lower *LSP*, *P*_max_, and *AQY*, but higher *LCP* and Rd compared to diploid species (Table 1). Two-way ANOVA results revealed that ploidy posed extremely significant effects on *LSP*, *P*_max_, *LCP*, *AQY*, and *R*_d_ of *Fragaria* species, while haplotype posed extremely significant effects on *LSP*, and the interactive effect between ploidy and haplotype had extremely significant effect on *P*_max_, and a significant effect on *R*_d_ (Table 2).

**Table 1:** Characteristics of light-response curves of *Fragaria* plants with different ploidy levels and haplotypes. The data are presented as mean ± standard deviation. Different lower case letters indicate significant differences between diploid and tetraploid level in the same haplotype at a *p* < 0.05 level.

Haplotype	Ploidy	Light saturation point (μmol·m^–2^·s^–1^)	*P*_*max*_ (μmol CO_2_·m^–2^·s^–1^)	Light compensation point (μmol·m^–2^·s^–1^)	Apparent quantum efficiency	Dark respiration rate (μmol CO_2_·m^–2^·s^–1^)
A	2	1122±26a	14.81±0.53a	9.47±1.03b	0.0377±0.0027a	0.88±0.07b
A	4	1048±22b	9.70±0.25b	13.92±1.67a	0.0303±0.0015b	1.04±0.05a
B	2	1070±32a	12.52±0.70a	8.56±0.96b	0.0365±0.0025a	0.69±0.05b
B	4	994±24b	10.85±0.36b	12.32±1.25a	0.0316±0.0011b	1.07±0.11a

**Table 2:** Effects of ploidy, haplotype, population (nested to haplotype), and interactive ploidy × haplotype on the characteristics of light-response curves of *Fragaria*. Values in bold indicate significant effect at a *p* < 0.05 level.

Factor	Light saturation point		*P*_*max*_		Light compensation point		Apparent quantum yield		Dark respiration rate	
	*F*	*P*	*F*	*P*	*F*	*P*	*F*	*P*	*F*	*P*
Ploidy	**26.269**	**0.001**	**141.948**	**<0.001**	**35.851**	**<0.001**	**11.057**	**0.009**	**36.860**	**<0.001**
Haplotype	**12.917**	**0.006**	4.071	0.074	3.339	0.101	2.567	0.144	3.282	0.103
Ploidy × Haplotype	0.002	0.967	**36.625**	**<0.001**	0.251	0.628	0.513	0.492	**6.850**	**0.028**

### Characteristics of CO_2_-response curves

**Fig 2:**
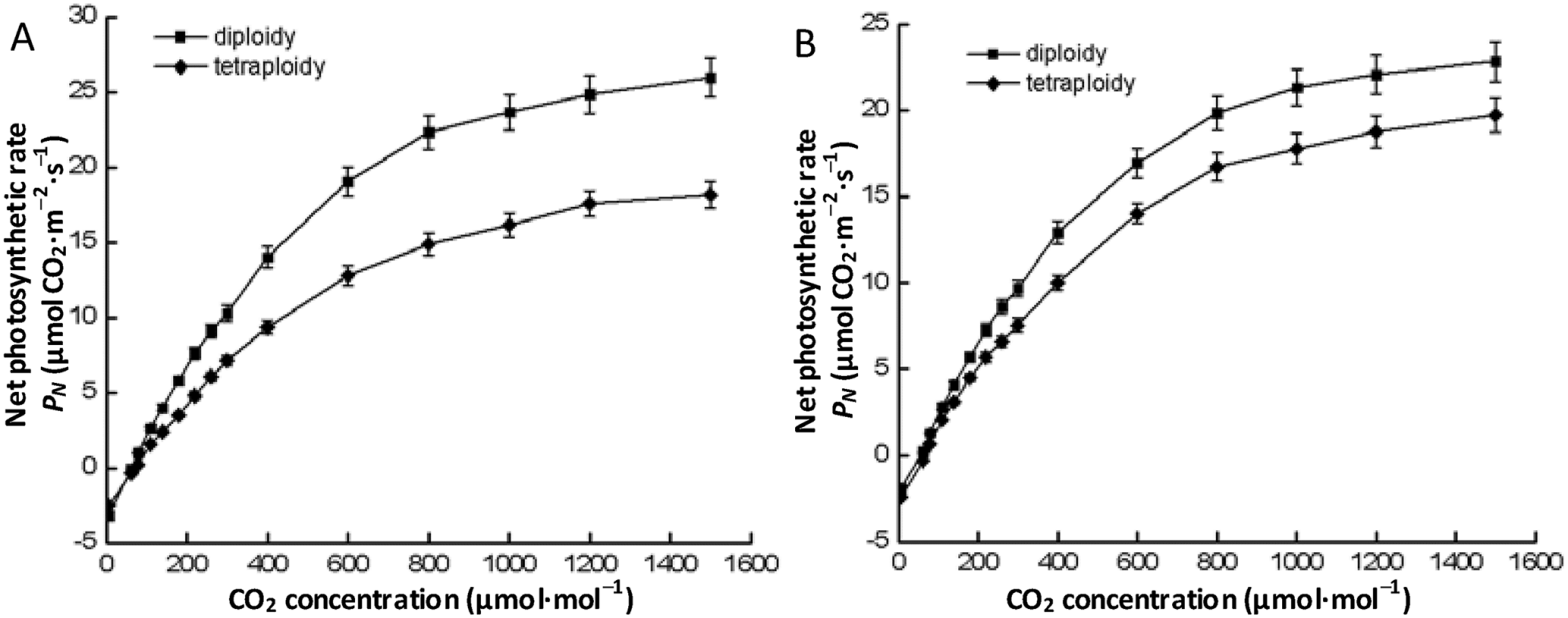
CO_2_-response curve of *Fragaria* plants with different ploidy level of haplotype A (A) and haplotype B (B).

The patterns of CO_2_-response curves differed among different ploidy levels (species) as well as among different haplotypes (Fig 2A and 2B, S3 Table). For identical haplotypes, tetraploid species had lower *CSP*, *P*_max_, and *ACE*, but higher *CCP* compared to diploid species (Table 3). Two-way ANOVAs results revealed that ploidy posed extremely significant effects on *CSP*, *P*_max_, and *ACE* of *Fragaria* species, and haplotype posed significant effects on *CS*P, while the interactive effect between ploidy and haplotype had no significant effect on these traits (Table 4).

**Table 3:** Characteristics of CO_2_-response curves of *Fragaria* with different ploidy levels in haplotype A and haplotype B. Different lower case letters indicate significant differences between diploid and tetraploid level in the same haplotype at a *p* < 0.05 level.

Haplotype	Ploidy	CO_2_ saturation point (μmol·mol^–1^)	CO_2_ compensation point (μmol·mol^–1^)	*P*_*max*_ (μmol CO_2_·m^–2^·s^–1^)	Apparent carboxylation efficiency
A	2	2253±114a	64.63±5.95b	27.04±1.95a	0.0522±0.0039a
A	4	1799±78b	78.12±6.92a	23.23±1.62b	0.0371±0.0031b
B	2	2015±99a	69.30±6.05b	25.96±0.70a	0.0503±0.0023a
B	4	1738±84b	76.75±8.67a	22.52±0.74b	0.0361±0.0034b

**Table 4:** Effects of ploidy, haplotype, population (nested to haplotype), and interactive ploidy × haplotype on the characteristics of CO_2_-response curves of *Fragaria*. Values in bold indicate significant effect at a *p* < 0.05 level.

Factor	CO_2_ saturation point		CO_2_ compensation point		*P*_*max*_		Apparent carboxylation efficiency	
	*F*	*P*	*F*	*P*	*F*	*P*	*F*	*P*
Ploidy	**47.964**	**<0.001**	3.812	0.083	**21.772**	**0.001**	**11.287**	**0.008**
Haplotype	**7.981**	**0.020**	0.095	0.765	0.807	0.392	0.073	0.794
Ploidy × Haplotype	2.806	0.128	0.318	0.587	0.001	0.979	0.000	0.986

### Characteristics of leaf gas exchange

**Fig 3:**
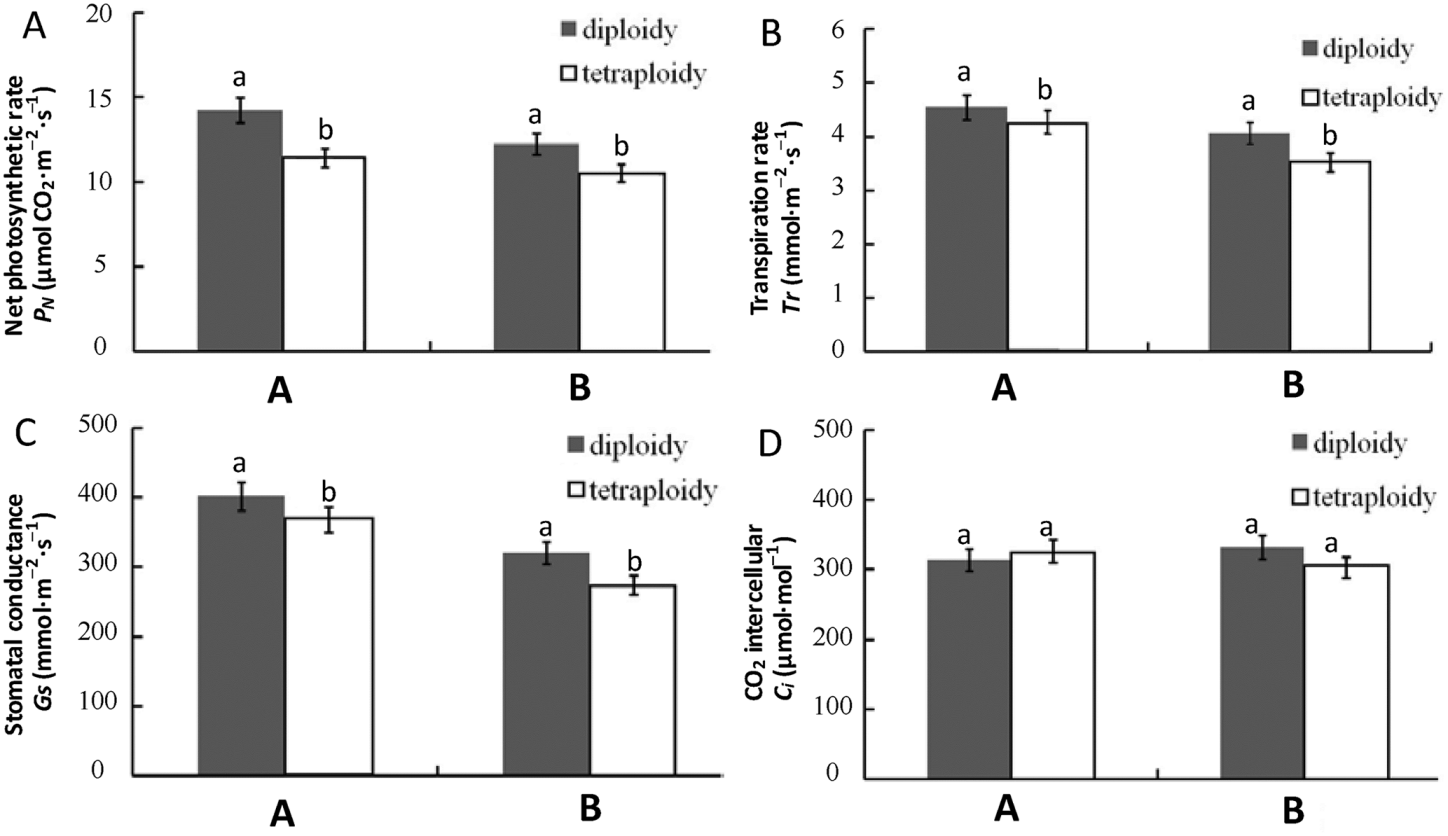
Net photosynthetic rate (A), transpiration rate (B), stomatal conductance (C), and intercellular CO_2_ concentration (D) of *Fragaria* plants with different ploidy levels and haplotypes.

For identical haplotypes, mean *P*_n_, *T*_r_, and *G*_s_ of tetraploid species were significantly higher than for diploid species (Fig 3A, 3B and 3C, S4 Table). There was no significant difference in the intercellular CO_2_ concentration between tetraploid and diploid species (Fig 3D, S4 Table). Two-way ANOVA results revealed that ploidy posed significant effects on *P*_n_, *T*_r_, and *G*_s_ of *Fragaria* species, while haplotype posed a significant effect on *T*_r_, *G*_s_, and *C*_i_, and no significant interactive effect was detected (Table 5). Most of the variance existed among individuals within haplotypes, while little variance existed among ploidy levels and haplotypes (Table 6).

**Table 5:** Two-way ANOVA analysis of ploidy, haplotype, and interactive ploidy and haplotype on the photosynthetic characteristics of *Fragaria*. Values in bold indicate significant effect at a *p* < 0.05 level.

Factor	Net photosynthetic rate		Transpiration rate		Stomatal conductance		Intercellular CO_2_ concentration	
	*F*	*P*	*F*	*P*	*F*	*P*	*F*	*P*
Ploidy	**7.627**	**0.006**	**14.262**	**<0.001**	**19.917**	**<0.001**	0.241	0.624
Haplotype	0.959	0.329	**10.287**	**0.002**	**4.345**	**0.039**	**5.734**	**0.018**
Ploidy × Haplotype	0.102	0.750	0.354	0.553	0.058	0.811	0.046	0.830

**Table 6:** Components of the variance of photosynthetic characteristics of *Fragaria* plants among ploidy levels (species), among haplotypes and within haplotypes.

	Net photosynthetic rate	Transpiration rate	Stomatal conductance	Intercellular CO_2_ concentration
Among ploidy levels	6.0%	9.0%	8.0%	0.0%
Among haplotypes	1.0%	6.0%	3.0%	3.0%
Within haplotypes	93.0%	85.0%	89.0%	97.0%

## Discussion

Polyploidy has major evolutionary significance, especially in plants [34]. Our results revealed that ploidy had significant effects on the characteristics of light-response curves, CO_2_-response curves, and leaf gas exchange parameters, with the exception of the intercellular CO_2_ concentration. Similar results have also been reported for the *Triticum* genus [16]. These results suggest polyploidization might play an important role in the evolution of photosynthesis of *Fragaria* species with increasing ploidy level, i.e. from diploid to tetraploid *Fragaria* species. Our results positively supported the previous studies showing that polyploidy has distinct effects on photosynthesis [15, 35].

Polyploidy caused unique photosynthetic characteristics that evidently differ from those of diploid varieties due to complex changes in anatomical traits and biochemical responses [13, 36, 37]. Both *LSP* and *LCP* of plants reflect the requirement of the light condition of the plant and are taken as the indicators to acquiring light energy [38]. Our results revealed that diploid species had higher *LSP*, but lower *LCP* compared to tetraploid species, indicating that diploid *F*. *pentaphylla* could utilize a higher level of *PAR* and thus adapt to higher light illumination intensities than tetraploid *F*. *moupinensis* [39]. This lower *LSP* and higher *LCP* might reveal an adaptive evolutionary trait of plants under higher irradiance [40]. Based on Maxent modeling, we found that the niche breadth of diploid *Fragaria* plants was considerably larger than that of tetraploid *Fragaria* plants and that the tetraploid plants were likely to grow in regions of higher altitude (unpublished data). Furthermore, *F*. *moupinensis* is most often found in the undergrowth of forests, mountain meadows, and grasslands at higher elevations [26]. Thus, the lower *LSP* and higher *LCP* might be an important trait of tetraploid *F*. *moupinensis* to adapt to shady light environments of high altitude. These results also indicated that polyploidization shrinks the adaptive region of *Fragaria* to light intensities. The higher *LCP* and *Rd* of tetraploid species compared to dipoloid species also indicated that tetraploid *Fragaria* species consumed more photosynthetic accumulations to maintain a normal physiological metabolism, adapted to shady light environments at high altitude.

CO_2_ is the most important substrate within photosynthesis [41]. The polyploidization effect on *CSP* has been found for tetraploid poplars, which were evidently lower than those of diploid poplar clones [42]. Similarly, in our study, we found tetraploid species to have lower *CSP*, *P*_max_, and *ACE* than diploid species, but higher *CCP*, indicating tetraploid *Fragaria* have a smaller adaptive region to CO_2_ concentration and a weak ability to utilize low CO_2_ concentrations [39]. The *CCP* ranges from 30 to 70 μmol·mol^–1^ in common plants [42]. In this study, the *CCP* of both tetraploid and diploid species varied from 53 to 86 μmol·mol^–1^, indicating that the ability of both *Fragaria* species to use CO_2_ were relatively low. The lower *LSP* and higher *CCP* values might be an important trait of *F*. *moupinensis* to adapt to lowered CO_2_ concentrations at high altitude.

Our results revealed significantly higher mean *P*_n_, *T*_r_, and *G*_s_ of diploid species compared to tetraploid species, indicating polypoidy had a negative effect on the photosynthetic capacity of *Fragaria* species. Several studies on the photosynthetic capacity of various ploidy levels illustrated that the *P*_*n*_ per unit cell basis decreased along with an increase in ploidy level [15, 16, 19, 43, 44]; e.g., in citrus trees, the net CO_2_ assimilation rates based on leaves in autotetraploids were 24–35% lower than for their diploid counterparts [19]. Srivalli and Khanna-Chopra reported that polyploidy enlarged the mesophylle cells of leaves, thus reducing the surface area to volume ratio of the cells, providing increased resistance for CO_2_ exchange [43]. Li et al. reported that polyploidy enlarged the stomata and decreased stomata density, resulting in decreasing *G*_s_, followed by *T*_r_, which consequently slowed the photosynthetic system, leading to slower *C*_i_ absorption and consequently less production [44]. Srivalli and Khanna-Chopra also reported that polyploidy reduced the ribulose-1,5-bisphosphate carboxylase/oxygenase (RuBPCO) content, thus reducing the rate of N mobilization, leading to lower *P*_n_ [43]. On the contrary, Austin et al. found that polyploidy can increased the ratio of chlorophyll a/b, i.e. led to a higher concentration of photosystems per chlorophyll, thus resulting in a higher photosynthetic rate per cell [45]. The conflicts indicated that more studies on more species are required to draw a general rule of the effect of polyploidization. In addition, it is important to understand the effect of polyploidy on morphological and physiological traits of plants, which can explain the mechanisms and ecological consequences that polyploidy offers [35, 46]. Further comparative studies should be conducted to concern these traits between diploidy and tetraploidy *Fragaria* species with same haplotypes in order to explore the adaptive mechanisms underlying the polyploidization.

Recently, the origin of whole genome was verified to play important roles in determining the effect of polyploidization [47]. Bilgrami et al. found that the whole chromosomes (genome) origin had a significant effect on *P*_n_, *T*_r_, *G*_s_, and *C*_i_ of wheat during developmental phases [16]. Coate et al. found that *Glycine dolichocarpa* accessions with different origins indicated with different plastid types responded differently to plolyploidization [35]. In this study, chroloplast haplotypes were used to indicate the origins of the maternal ancestry [48], and we found significant effects of haplotype on *LSP*, *CSP*, *T*_r_, and *C*_i_, as well as a significant interactive effect of ploidy and haplotype on *P*_max_ and *R*_d_ of the light-response curve. These results indicated that the origin of the chromosome could also affect the photosynthetic characteristics of *Fragaria* species and even the polyploidization effect of photosynthetic traits. Our results further confirmed previous report that synthetic strawberry (*Fragaria*) allo-octoploids show varying photosynthetic responses depending on parental species combinations [49]. Furthermore, we found that most of the variances existed within haplotypes among individuals (genotypes), which distributed in different geographical sites, indicating a large local evolutionary adaptive potential of the photosynthetic traits in adaptation to heterogeous environments of *Fragaria* species [50]. Geographical and climatic factors have been recognized to be the most important selective force driving for the variation of phenotypic traits [51]. Several studies verified strong similarities in bioclimatic niches and functional traits of *Leucadendron* L. [52] and *Protea* L. [53] based on niche modeling. The present distribution patterns of both *Fragaria* species might be a result of long-term expansion after polyploidization [54].

In summary, the lower *LSP* and *CSP*, but higher *LCP*, *Rd*, and *CCP* of tetraploid *Fragaria* species compared to diploid species evolved as long-term adaption to the shady light environment of high altitude after the polyploidization. Such polyploidization might be the main driver for the evolution of photosynthetic characteristics of *Fragaria* species. Except for chromosome number, the origin of the whole chromosomes could also affect the photosynthetic characteristics of *Fragaria* species. Future studies should be aimed at discriminating the effect of the origin of the whole chromosomes, genotypes, the geographical factors, and climatic factors on phenotypic and functional traits of *Fragaria*.

## Supporting information

S1 Fig Sequences of ndhA gene in haplotype A and haplotype B. (TIF)

S1 Table The sample information of 175 individuals used in this experiment. (DOCX)

S2 Table The values of the means and standard deviations used to build graphs Fig 1. (DOCX)

S3 Table The values of the means and standard deviations used to build graphs Fig 2. (DOCX)

S4 Table The values of the means and standard deviations used to build graphs Fig 3. (DOCX)
