# Supplementary figures and images for: Effects of ploidy level and haplotype on variation of photosynthetic traits: Novel evidence from two Fragaria species
Source: PLoS One. 2017 Jun 23;12(6):e0179899. doi: 10.1371/journal.pone.0179899 (PMC5482484; doi:10.1371/journal.pone.0179899)

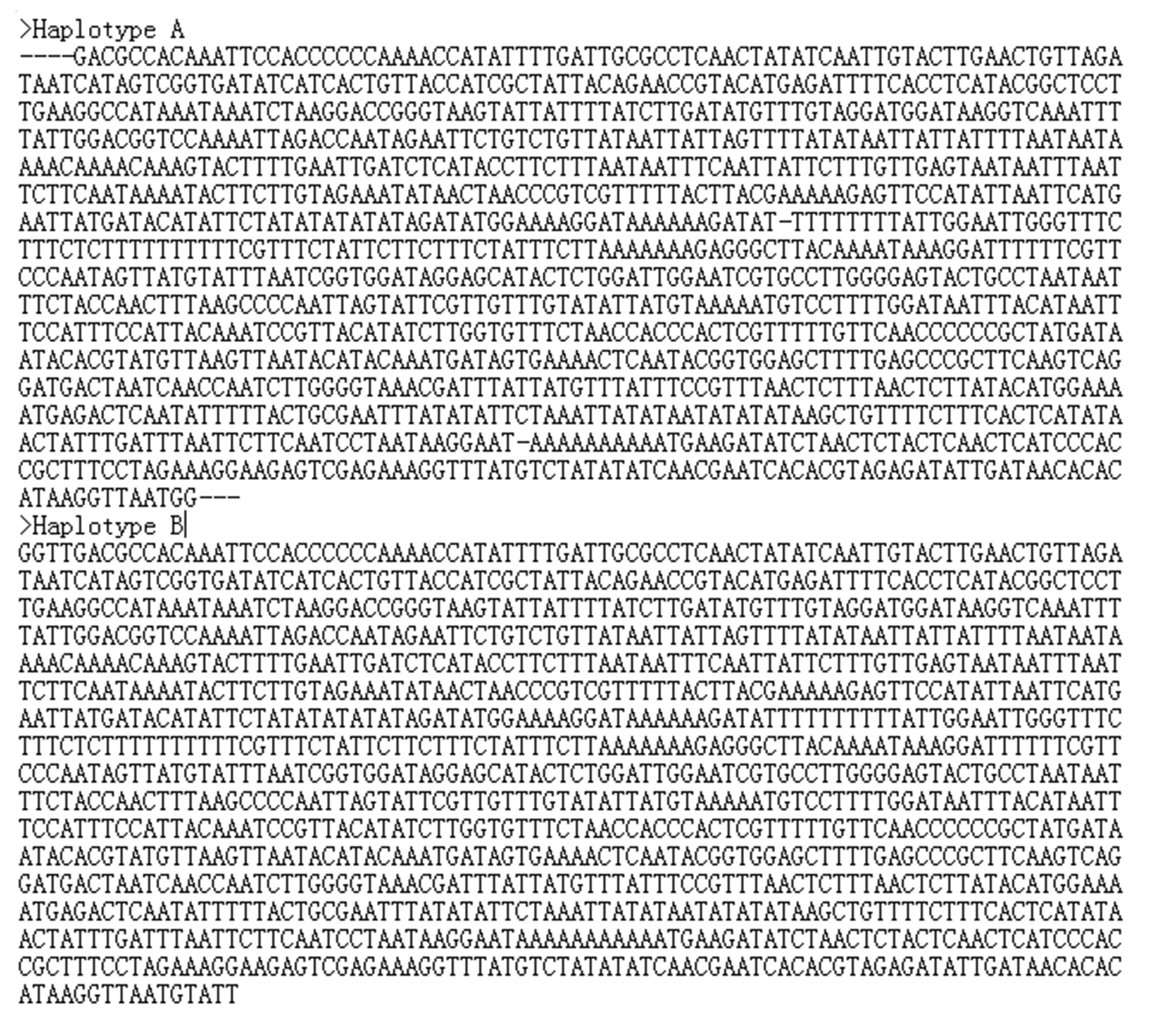

Supplement: S1 Fig — (TIF) [file pone.0179899.s001.tif]
