# Supplementary material for: Effects of ploidy level and haplotype on variation of photosynthetic traits: Novel evidence from two Fragaria species
Source: PLoS One. 2017 Jun 23;12(6):e0179899. doi: 10.1371/journal.pone.0179899 (PMC5482484; doi:10.1371/journal.pone.0179899)
Supplement: S1 Table — (DOCX) [file pone.0179899.s002.docx]

S1 Table The sample information of 175 individuals used in this experiment

| **No.** | **Province** | **Population No.** | **Individual No.** | **Latitude**  **(°E)** | **Longitude (°N)** | **Haplotype** | **Ploidy level** | **Species** |
| --- | --- | --- | --- | --- | --- | --- | --- | --- |
| 1 | Gansu | 1 | 1 | 33.3873 | 105.5090 | A | 2 | *Fragaria pentaphylla* |
| 2 | Gansu | 2 | 1 | 35.7757 | 103.9640 | A | 4 | *Fragaria moupinensis* |
| 3 | Gansu | 3 | 1 | 35.8345 | 104.1240 | A | 2 | *Fragaria pentaphylla* |
| 4 | Gansu | 3 | 2 |  |  | A | 2 | *Fragaria pentaphylla* |
| 5 | Gansu | 3 | 3 |  |  | A | 2 | *Fragaria pentaphylla* |
| 6 | Gansu | 3 | 4 |  |  | A | 2 | *Fragaria pentaphylla* |
| 10 | Gansu | 3 | 5 |  |  | A | 2 | *Fragaria pentaphylla* |
| 11 | Gansu | 4 | 1 | 34.7095 | 102.9990 | A | 2 | *Fragaria pentaphylla* |
| 12 | Gansu | 4 | 2 |  |  | A | 2 | *Fragaria pentaphylla* |
| 13 | Gansu | 4 | 3 |  |  | A | 4 | *Fragaria moupinensis* |
| 14 | Gansu | 5 | 1 | 34.4341 | 102.5940 | A | 2 | *Fragaria pentaphylla* |
| 15 | Gansu | 6 | 1 | 34.4228 | 103.0100 | A | 2 | *Fragaria pentaphylla* |
| 16 | Gansu | 7 | 1 | 34.7025 | 103.0240 | A | 2 | *Fragaria pentaphylla* |
| 17 | Gansu | 7 | 2 |  |  | A | 2 | *Fragaria pentaphylla* |
| 18 | Gansu | 7 | 3 |  |  | A | 2 | *Fragaria pentaphylla* |
| 19 | Gansu | 7 | 4 |  |  | A | 2 | *Fragaria pentaphylla* |
| 20 | Gansu | 8 | 1 | 33.0683 | 104.7380 | A | 2 | *Fragaria pentaphylla* |
| 21 | Gansu | 9 | 1 | 33.2238 | 104.7250 | A | 2 | *Fragaria pentaphylla* |
| 22 | Gansu | 10 | 1 | 33.3387 | 104.8570 | A | 4 | *Fragaria moupinensis* |
| 23 | Gansu | 11 | 1 | 33.5704 | 105.0950 | A | 2 | *Fragaria pentaphylla* |
| 24 | Gansu | 12 | 1 | 33.3823 | 105.5300 | A | 2 | *Fragaria pentaphylla* |
| 25 | Gansu | 13 | 1 | 34.938 | 102.9170 | A | 2 | *Fragaria pentaphylla* |
| 26 | Gansu | 14 | 1 | 34.8349 | 103.0010 | A | 4 | *Fragaria moupinensis* |
| 27 | Gansu | 15 | 1 | 34.8239 | 103.1310 | A | 4 | *Fragaria moupinensis* |
| 28 | Gansu | 16 | 1 | 34.9424 | 102.8900 | A | 2 | *Fragaria pentaphylla* |
| 29 | Qinghai | 1 | 1 | 36.6345 | 101.7490 | A | 2 | *Fragaria pentaphylla* |
| 30 | Qinghai | 1 | 4 |  |  | A | 4 | *Fragaria moupinensis* |
| 31 | Qinghai | 2 | 1 | 36.9387 | 101.7020 | A | 2 | *Fragaria pentaphylla* |
| 32 | Qinghai | 3 | 1 | 37.3586 | 101.8210 | A | 2 | *Fragaria pentaphylla* |
| 33 | Qinghai | 3 | 2 |  |  | A | 2 | *Fragaria pentaphylla* |
| 34 | Qinghai | 3 | 3 |  |  | A | 2 | *Fragaria pentaphylla* |
| 35 | Qinghai | 3 | 4 |  |  | A | 2 | *Fragaria pentaphylla* |
| 36 | Qinghai | 3 | 5 |  |  | A | 2 | *Fragaria pentaphylla* |
| 37 | Qinghai | 4 | 1 | 37.3614 | 101.7040 | A | 2 | *Fragaria pentaphylla* |
| 38 | Qinghai | 5 | 1 | 36.9844 | 102.4330 | A | 2 | *Fragaria pentaphylla* |
| 39 | Qinghai | 5 | 1 | 36.9844 | 102.4330 | A | 2 | *Fragaria pentaphylla* |
| 40 | Qinghai | 6 | 1 | 36.9146 | 102.3640 | A | 2 | *Fragaria pentaphylla* |
| 41 | Qinghai | 6 | 2 |  |  | A | 2 | *Fragaria pentaphylla* |
| 42 | Qinghai | 6 | 3 |  |  | A | 4 | *Fragaria moupinensis* |
| 43 | Qinghai | 7 | 1 | 35.8796 | 102.4390 | A | 2 | *Fragaria pentaphylla* |
| 44 | Qinghai | 7 | 1 |  |  | A | 2 | *Fragaria pentaphylla* |
| 45 | Qinghai | 7 | 2 |  |  | A | 2 | *Fragaria pentaphylla* |
| 46 | Qinghai | 7 | 3 |  |  | A | 2 | *Fragaria pentaphylla* |
| 47 | Qinghai | 7 | 4 |  |  | A | 2 | *Fragaria pentaphylla* |
| 48 | Qinghai | 8 | 1 | 36.2805 | 101.6790 | A | 4 | *Fragaria moupinensis* |
| 49 | Qinghai | 8 | 2 |  |  | A | 2 | *Fragaria pentaphylla* |
| 50 | Qinghai | 9 | 1 | 36.6699 | 101.3660 | A | 2 | *Fragaria pentaphylla* |
| 51 | Qinghai | 9 | 2 |  |  | A | 2 | *Fragaria pentaphylla* |
| 52 | Qinghai | 9 | 3 |  |  | A | 2 | *Fragaria pentaphylla* |
| 53 | Qinghai | 9 | 4 |  |  | A | 2 | *Fragaria pentaphylla* |
| 54 | Qinghai | 9 | 5 |  |  | A | 2 | *Fragaria pentaphylla* |
| 55 | Qinghai | 10 | 1 | 36.6590 | 101.4020 | A | 2 | *Fragaria pentaphylla* |
| 56 | Qinghai | 11 | 1 | 36.8692 | 101.3630 | A | 2 | *Fragaria pentaphylla* |
| 57 | Qinghai | 11 | 2 |  |  | A | 2 | *Fragaria pentaphylla* |
| 58 | Qinghai | 11 | 3 |  |  | A | 2 | *Fragaria pentaphylla* |
| 59 | Qinghai | 11 | 4 |  |  | A | 4 | *Fragaria moupinensis* |
| 60 | Qinghai | 12 | 1 | 36.6588 | 101.4850 | A | 2 | *Fragaria pentaphylla* |
| 61 | Qinghai | 12 | 2 |  |  | A | 2 | *Fragaria pentaphylla* |
| 62 | Qinghai | 13 | 1 | 36.1785 | 102.6620 | A | 2 | *Fragaria pentaphylla* |
| 63 | Qinghai | 14 | 1 | 36.1735 | 102.6430 | A | 2 | *Fragaria pentaphylla* |
| 64 | Qinghai | 15 | 1 | 36.1718 | 102.6370 | A | 2 | *Fragaria pentaphylla* |
| 65 | Qinghai | 15 | 2 |  |  | A | 2 | *Fragaria pentaphylla* |
| 66 | Qinghai | 15 | 3 |  |  | A | 2 | *Fragaria pentaphylla* |
| 67 | Qinghai | 15 | 4 |  |  | A | 2 | *Fragaria pentaphylla* |
| 7 | Qinghai | 16 | 1 | 35.8018 | 102.6830 | A | 2 | *Fragaria pentaphylla* |
| 8 | Qinghai | 16 | 2 |  |  | A | 2 | *Fragaria pentaphylla* |
| 9 | Qinghai | 16 | 3 |  |  | A | 2 | *Fragaria pentaphylla* |
| 71 | Shaanxi | 1 | 1 | 33.2786 | 108.3020 | A | 2 | *Fragaria pentaphylla* |
| 72 | Shaanxi | 2 | 1 | 33.2743 | 108.2700 | A | 2 | *Fragaria pentaphylla* |
| 73 | Shaanxi | 2 | 2 |  |  | A | 2 | *Fragaria pentaphylla* |
| 74 | Shaanxi | 2 | 3 |  |  | A | 2 | *Fragaria pentaphylla* |
| 75 | Shaanxi | 3 | 1 | 34.0093 | 107.4860 | A | 2 | *Fragaria pentaphylla* |
| 76 | Shaanxi | 4 | 1 | 33.5479 | 107.4750 | A | 2 | *Fragaria pentaphylla* |
| 77 | Shaanxi | 4 | 2 |  |  | A | 2 | *Fragaria pentaphylla* |
| 78 | Sichuan | 1 | 1 | 31.6878 | 103.8780 | A | 2 | *Fragaria pentaphylla* |
| 79 | Sichuan | 1 | 2 |  |  | A | 2 | *Fragaria pentaphylla* |
| 80 | Sichuan | 1 | 1 |  |  | A | 4 | *Fragaria moupinensis* |
| 81 | Sichuan | 2 | 2 | 31.6602 | 102.7830 | A | 2 | *Fragaria pentaphylla* |
| 82 | Sichuan | 2 | 3 |  |  | A | 4 | *Fragaria moupinensis* |
| 83 | Sichuan | 2 | 1 |  |  | B | 4 | *Fragaria moupinensis* |
| 84 | Sichuan | 3 | 1 | 31.1611 | 103.5810 | A | 2 | *Fragaria pentaphylla* |
| 85 | Sichuan | 3 | 2 |  |  | A | 4 | *Fragaria moupinensis* |
| 86 | Sichuan | 3 | 1 |  |  | B | 2 | *Fragaria pentaphylla* |
| 87 | Sichuan | 4 | 1 | 31.8833 | 102.2170 | A | 2 | *Fragaria pentaphylla* |
| 88 | Sichuan | 4 | 2 |  |  | A | 2 | *Fragaria pentaphylla* |
| 89 | Sichuan | 4 | 3 |  |  | A | 2 | *Fragaria pentaphylla* |
| 90 | Sichuan | 4 | 4 |  |  | A | 2 | *Fragaria pentaphylla* |
| 91 | Sichuan | 4 | 5 |  |  | A | 2 | *Fragaria pentaphylla* |
| 92 | Sichuan | 4 | 6 |  |  | A | 4 | *Fragaria moupinensis* |
| 93 | Sichuan | 4 | 2 |  |  | B | 2 | *Fragaria pentaphylla* |
| 94 | Sichuan | 5 | 1 | 31.8167 | 102.6670 | A | 2 | *Fragaria pentaphylla* |
| 95 | Sichuan | 5 | 2 |  |  | A | 2 | *Fragaria pentaphylla* |
| 96 | Sichuan | 5 | 3 |  |  | A | 2 | *Fragaria pentaphylla* |
| 97 | Sichuan | 5 | 4 |  |  | A | 2 | *Fragaria pentaphylla* |
| 98 | Sichuan | 5 | 5 |  |  | A | 4 | *Fragaria moupinensis* |
| 99 | Sichuan | 6 | 1 | 29.8414 | 102.0440 | A | 2 | *Fragaria pentaphylla* |
| 100 | Sichuan | 6 | 2 |  |  | A | 2 | *Fragaria pentaphylla* |
| 101 | Sichuan | 7 | 1 | 32.6617 | 104.6880 | A | 2 | *Fragaria pentaphylla* |
| 102 | Sichuan | 7 | 2 |  |  | A | 2 | *Fragaria pentaphylla* |
| 103 | Sichuan | 7 | 3 |  |  | A | 2 | *Fragaria pentaphylla* |
| 104 | Sichuan | 7 | 4 |  |  | A | 2 | *Fragaria pentaphylla* |
| 105 | Sichuan | 7 | 5 |  |  | A | 2 | *Fragaria pentaphylla* |
| 106 | Sichuan | 8 | 1 | 31.2497 | 102.8870 | A | 2 | *Fragaria pentaphylla* |
| 107 | Sichuan | 8 | 2 |  |  | A | 2 | *Fragaria pentaphylla* |
| 108 | Sichuan | 9 | 1 | 32.5791 | 104.6660 | A | 4 | *Fragaria moupinensis* |
| 109 | Sichuan | 10 | 1 | 32.5768 | 104.6700 | A | 2 | *Fragaria pentaphylla* |
| 110 | Sichuan | 10 | 2 |  |  | A | 2 | *Fragaria pentaphylla* |
| 111 | Sichuan | 10 | 3 |  |  | A | 2 | *Fragaria pentaphylla* |
| 112 | Sichuan | 10 | 4 |  |  | A | 2 | *Fragaria pentaphylla* |
| 113 | Sichuan | 11 | 1 | 29.9147 | 101.9920 | B | 2 | *Fragaria pentaphylla* |
| 114 | Sichuan | 12 | 1 | 29.9272 | 101.9800 | B | 4 | *Fragaria moupinensis* |
| 115 | Sichuan | 13 | 1 | 30.0175 | 101.8590 | B | 4 | *Fragaria moupinensis* |
| 116 | Sichuan | 13 | 2 |  |  | B | 4 | *Fragaria moupinensis* |
| 117 | Sichuan | 13 | 3 |  |  | B | 4 | *Fragaria moupinensis* |
| 118 | Sichuan | 14 | 1 | 30.0432 | 101.8270 | B | 2 | *Fragaria pentaphylla* |
| 119 | Sichuan | 14 | 1 |  |  | B | 4 | *Fragaria moupinensis* |
| 120 | Sichuan | 15 | 1 | 30.0071 | 101.7760 | B | 4 | *Fragaria moupinensis* |
| 121 | Sichuan | 15 | 2 |  |  | B | 4 | *Fragaria moupinensis* |
| 122 | Sichuan | 15 | 3 |  |  | B | 4 | *Fragaria moupinensis* |
| 123 | Sichuan | 15 | 4 |  |  | B | 4 | *Fragaria moupinensis* |
| 124 | Sichuan | 15 | 5 |  |  | B | 4 | *Fragaria moupinensis* |
| 125 | Xizang | 1 | 1 | 29.5639 | 94.5109 | A | 4 | *Fragaria moupinensis* |
| 126 | Xizang | 1 | 1 |  |  | B | 2 | *Fragaria pentaphylla* |
| 127 | Xizang | 1 | 2 |  |  | B | 2 | *Fragaria pentaphylla* |
| 128 | Xizang | 1 | 3 |  |  | B | 2 | *Fragaria pentaphylla* |
| 129 | Xizang | 1 | 4 |  |  | B | 4 | *Fragaria moupinensis* |
| 130 | Xizang | 1 | 5 |  |  | B | 4 | *Fragaria moupinensis* |
| 131 | Xizang | 1 | 6 |  |  | B | 4 | *Fragaria moupinensis* |
| 132 | Xizang | 2 | 1 | 29.5594 | 94.5578 | A | 4 | *Fragaria moupinensis* |
| 133 | Xizang | 2 | 2 |  |  | A | 2 | *Fragaria pentaphylla* |
| 134 | Xizang | 2 | 3 |  |  | A | 2 | *Fragaria pentaphylla* |
| 135 | Xizang | 2 | 4 |  |  | B | 4 | *Fragaria moupinensis* |
| 136 | Xizang | 2 | 5 |  |  | B | 4 | *Fragaria moupinensis* |
| 137 | Xizang | 2 | 6 |  |  | B | 2 | *Fragaria pentaphylla* |
| 138 | Xizang | 2 | 7 |  |  | B | 2 | *Fragaria pentaphylla* |
| 139 | Xizang | 2 | 8 |  |  | B | 2 | *Fragaria pentaphylla* |
| 140 | Xizang | 3 | 1 | 29.6022 | 94.6071 | A | 4 | *Fragaria moupinensis* |
| 141 | Xizang | 3 | 1 | 29.2190 | 94.2212 | B | 2 | *Fragaria pentaphylla* |
| 142 | Xizang | 4 | 1 | 29.67460 | 94.72620 | A | 4 | *Fragaria moupinensis* |
| 143 | Xizang | 4 | 2 |  |  | A | 2 | *Fragaria pentaphylla* |
| 144 | Xizang | 5 | 1 | 29.0542 | 92.4328 | A | 4 | *Fragaria moupinensis* |
| 145 | Xizang | 5 | 1 |  |  | B | 4 | *Fragaria moupinensis* |
| 146 | Xizang | 5 | 2 |  |  | B | 4 | *Fragaria moupinensis* |
| 147 | Xizang | 5 | 3 |  |  | B | 2 | *Fragaria pentaphylla* |
| 148 | Xizang | 5 | 4 |  |  | B | 2 | *Fragaria pentaphylla* |
| 149 | Xizang | 5 | 5 |  |  | B | 2 | *Fragaria pentaphylla* |
| 150 | Xizang | 6 | 1 | 29.0947 | 93.4311 | B | 4 | *Fragaria moupinensis* |
| 151 | Xizang | 6 | 1 |  |  | B | 2 | *Fragaria pentaphylla* |
| 152 | Xizang | 6 | 2 |  |  | B | 2 | *Fragaria pentaphylla* |
| 153 | Xizang | 6 | 3 |  |  | B | 2 | *Fragaria pentaphylla* |
| 154 | Xizang | 6 | 4 |  |  | B | 2 | *Fragaria pentaphylla* |
| 155 | Xizang | 6 | 5 |  |  | B | 4 | *Fragaria moupinensis* |
| 156 | Xizang | 6 | 6 |  |  | B | 4 | *Fragaria moupinensis* |
| 157 | Xizang | 7 | 1 | 29.6531 | 94.3823 | B | 2 | *Fragaria pentaphylla* |
| 158 | Xizang | 7 | 2 |  |  | B | 2 | *Fragaria pentaphylla* |
| 159 | Xizang | 7 | 3 |  |  | B | 2 | *Fragaria pentaphylla* |
| 160 | Xizang | 8 | 1 | 29.2190 | 94.2212 | B | 4 | *Fragaria moupinensis* |
| 161 | Xizang | 9 | 1 | 29.9542 | 94.8366 | B | 2 | *Fragaria pentaphylla* |
| 162 | Xizang | 10 | 1 | 29.6650 | 94.7186 | B | 4 | *Fragaria moupinensis* |
| 163 | Xizang | 10 | 2 |  |  | B | 4 | *Fragaria moupinensis* |
| 164 | Xizang | 11 | 1 | 29.6194 | 94.7086 | B | 4 | *Fragaria moupinensis* |
| 165 | Yunnan | 1 | 1 | 27.0355 | 100.2040 | B | 2 | *Fragaria pentaphylla* |
| 166 | Yunnan | 1 | 2 |  |  | B | 2 | *Fragaria pentaphylla* |
| 167 | Yunnan | 1 | 3 |  |  | B | 2 | *Fragaria pentaphylla* |
| 168 | Yunnan | 1 | 4 |  |  | B | 4 | *Fragaria moupinensis* |
| 169 | Yunnan | 1 | 5 |  |  | B | 4 | *Fragaria moupinensis* |
| 170 | Yunnan | 1 | 6 |  |  | B | 4 | *Fragaria moupinensis* |
| 171 | Yunnan | 2 | 1 | 27.5813 | 99.8277 | B | 2 | *Fragaria pentaphylla* |
| 172 | Yunnan | 3 | 1 | 27.3823 | 99.8833 | B | 4 | *Fragaria moupinensis* |
| 173 | Yunnan | 3 | 2 |  |  | B | 4 | *Fragaria moupinensis* |
| 174 | Yunnan | 3 | 3 |  |  | B | 2 | *Fragaria pentaphylla* |
| 175 | Yunnan | 3 | 4 |  |  | B | 2 | *Fragaria pentaphylla* |
