# Supplementary material for: Effects of ploidy level and haplotype on variation of photosynthetic traits: Novel evidence from two Fragaria species
Source: PLoS One. 2017 Jun 23;12(6):e0179899. doi: 10.1371/journal.pone.0179899 (PMC5482484; doi:10.1371/journal.pone.0179899)
Supplement: S2 Table — (DOCX) [file pone.0179899.s003.docx]

S2 Table The values of the means and standard deviations used to build graphs Fig 1

| Photosynthetic active radiation (μmol·m^–2^·s^–1^) | Haplotype A | | Haplotype B | |
| --- | --- | --- | --- | --- |
|  | Diploidy | Tetraploidy | Diploidy | Tetraploidy |
| 1500 | 14.93±0.95 | 9.56±0.48 | 10.87±0.70 | 9.86±1.08 |
| 1200 | 13.96±0.73 | 9.46±0.51 | 11.22±0.39 | 10.23±0.85 |
| 1000 | 13.52±0.68 | 9.74±0.35 | 11.38±0.22 | 10.54±0.43 |
| 800 | 12.93±0.65 | 8.88±0.46 | 11.23±0.22 | 9.47±0.38 |
| 600 | 12.33±0.47 | 8.19±0.35 | 11.08±0.29 | 8.28±0.54 |
| 400 | 10.40±0.38 | 7.37±0.38 | 9.24±0.46 | 7.47±0.40 |
| 300 | 8.46±0.33 | 6.99±0.44 | 8.56±0.30 | 6.59±0.21 |
| 250 | 7.54±0.38 | 6.58±0.35 | 7.63±0.30 | 5.75±0.23 |
| 200 | 6.57±0.33 | 5.58±0.35 | 6.64±0.25 | 5.24±0.16 |
| 150 | 5.36±0.15 | 4.43±0.28 | 5.65±0.18 | 4.53±0.21 |
| 100 | 3.95±0.15 | 3.63±0.34 | 4.10±0.21 | 3.20±0.12 |
| 50 | 1.81±0.20 | 1.77±0.23 | 2.13±0.10 | 1.25±0.07 |
| 20 | 0.37±0.14 | 0.62±0.21 | 0.27±0.13 | 0.51±0.12 |
| 0 | -0.84±0.11 | -0.68±0.29 | -0.68±0.16 | -0.71±0.07 |
