# Supplementary material for: Effects of ploidy level and haplotype on variation of photosynthetic traits: Novel evidence from two Fragaria species
Source: PLoS One. 2017 Jun 23;12(6):e0179899. doi: 10.1371/journal.pone.0179899 (PMC5482484; doi:10.1371/journal.pone.0179899)
Supplement: S3 Table — (DOCX) [file pone.0179899.s004.docx]

S3 Table The values of the means and standard deviations used to build graphs Fig 2

| CO_2_ concentration  (μmol·mol^-1^) | Haplotype A | | Haplotype B | |
| --- | --- | --- | --- | --- |
|  | Diploidy | Tetraploidy | Diploidy | Tetraploidy |
| 1500 | 26.28±0.72 | 17.59±0.53 | 22.86±0.99 | 19.06±0.81 |
| 1200 | 24.82±0.71 | 16.48±0.45 | 22.26±0.66 | 18.10±0.90 |
| 1000 | 23.07±0.65 | 15.62±0.26 | 21.41±0.60 | 17.20±0.69 |
| 800 | 22.43±0.61 | 14.96±0.30 | 19.35±0.77 | 16.44±0.54 |
| 600 | 19.32±0.56 | 12.07±0.39 | 16.67±0.57 | 13.20±0.38 |
| 400 | 14.27±0.58 | 8.46±0.34 | 13.53±0.43 | 9.74±0.45 |
| 300 | 10.21±0.47 | 7.18±0.30 | 9.50±0.19 | 7.22±0.42 |
| 250 | 8.74±0.42 | 5.41±0.33 | 8.46±0.28 | 6.51±0.27 |
| 200 | 7.26±0.49 | 3.62±0.27 | 6.69±0.30 | 5.23±0.23 |
| 150 | 5.72±0.25 | 3.21±0.29 | 5.33±0.14 | 3.70±0.13 |
| 100 | 4.26±0.25 | 2.44±0.33 | 3.19±0.09 | 1.21±0.17 |
| 50 | 2.62±0.27 | -0.32±0.26 | -0.07±0.03 | -0.14±0.06 |
| 20 | -0.38±0.24 | -0.43±0.08 | -0.33±0.17 | -0.91±0.07 |
| 0 | -3.15±0.12 | -2.25±0.24 | -2.72±0.30 | -2.85±0.08 |
