# Supplementary material for: Effects of ploidy level and haplotype on variation of photosynthetic traits: Novel evidence from two Fragaria species
Source: PLoS One. 2017 Jun 23;12(6):e0179899. doi: 10.1371/journal.pone.0179899 (PMC5482484; doi:10.1371/journal.pone.0179899)
Supplement: S4 Table — (DOCX) [file pone.0179899.s005.docx]

S4 Table The values of the means and standard deviations used to build graphs Fig 3

| Traits | Haplotype A | | Haplotype B | |
| --- | --- | --- | --- | --- |
|  | Diploidy | Tetraploidy | Diploidy | Tetraploidy |
| *P*_n_ (μmol CO_2_·m^–2^·s^–1^) | 14.64±1.40 | 10.40±0.84 | 12.78±0.75 | 10.26±0.65 |
| *T*_r_ (mmol·m^–2^·s^–1^) | 4.69±0.37 | 4.01±0.30 | 3.95±0.22 | 3.42±0.19 |
| *G*_s_ (mmol·m^–2^·s^–1^) | 399.84±28.64 | 361.79±17.32 | 310.74±20.51 | 269.61±19.27 |
| *C*_i_ (μmol·mol^–1^) | 309.26±21.09 | 311.85±22.39 | 310.13±27.27 | 302.18±19.38 |
